# Dynamic externalities of basic medical insurance compensation, health capital accumulation, and economic growth

**DOI:** 10.3389/fpubh.2025.1562417

**Published:** 2025-05-01

**Authors:** Xinyue Yuan, Xujin Yang

**Affiliations:** ^1^School of Journalism, Communication University of China, Beijing, China; ^2^School of Mathematics and Statistics, Hunan University of Technology and Business, Changsha, China

**Keywords:** basic medical insurance, health capital accumulation, economic growth, saddle path, subsidy

## Abstract

**Background:**

A strong healthcare system plays a vital role in reducing the financial burden on households while simultaneously promoting sustainable economic development. This study investigates how dynamic medical insurance compensation affects health capital accumulation and economic growth, thereby revealing the broader economic implications of health policy design.

**Methods:**

This paper develops a neoclassical economic growth model that incorporates dynamic basic medical insurance and health capital. Utilizing the shooting algorithm, we compute equilibrium solutions at each period along the saddle path and analyze the external effects of different compensation strategies. This approach enables a systematic evaluation of how varying the compensation rate influences welfare, labor allocation, and long-term growth trajectories.

**Results:**

The absence of medical insurance compensation or a constant compensation rate leads to a decline in welfare over time. In contrast, a progressively decreasing compensation rate significantly promotes the consumption of healthcare goods, increases health capital, and stimulates economic growth. The model also shows that this compensation design enhances overall welfare along the transition path and induces a partial reallocation of labor toward the healthcare sector.

**Conclusions:**

The findings highlight the importance of dynamic, policy-responsive compensation structures in maximizing long-term welfare. A gradually declining medical insurance compensation rate not only improves population health and boosts economic output but also provides a viable strategy for labor market optimization and health system sustainability. These insights offer valuable guidance for health policy reform in developing and transitioning economies.

## 1 Introduction

Over the past two decades, China’s healthcare expenditure has grown rapidly, placing a significant financial burden on individuals and households. Although China introduced the New Rural Cooperative Medical Scheme (NRCMS) in 2003 and the Urban Resident Basic Medical Insurance (URBMI) in 2007, and has gradually increased the reimbursement rate of Basic Medical Insurance (BMI) in recent years, the overall reimbursement rate remains relatively rigid and has not adequately adapted to the growing demand for health investment across different stages of economic development. Specifically, China’s current BMI system shows delays in adjusting reimbursement ratios, reforming financing structures, and expanding coverage scope. As a result, out-of-pocket healthcare expenditures remain high, and the financial burden on residents persists.

From an international perspective, countries with similar stages of economic development to China, such as Thailand, Brazil, and Mexico, have implemented phased and dynamic adjustments to their medical insurance reimbursement mechanisms and continuously expanded coverage. These efforts have significantly reduced individuals’ medical expenses and enhanced the accumulation of health capital and overall social welfare. For example, Thailand launched its Universal Coverage Scheme (UCS) in 2002, which nearly achieved full reimbursement of medical expenses, significantly reducing residents’ out-of-pocket payments. Brazil implemented a free healthcare policy through its Unified Health System (SUS) to ensure universal access to medical services. Mexico, meanwhile, established the national public health insurance program Seguro Popular in 2004, which significantly improved the accessibility and depth of public healthcare services within just over a decade. These countries have successfully tailored the intensity of medical security to their respective stages of economic development, thereby avoiding the burden caused by rigid reimbursement systems and achieving positive outcomes in both health and economic development.

In light of this, a pressing issue China faces is the need to establish a more flexible and effective dynamic adjustment mechanism for BMI reimbursement rates. This would accommodate rising health demands driven by increased income levels and alleviate the healthcare burden on residents. It would also prevent institutional rigidity from hindering the accumulation of health capital and long-term economic growth. This paper aims to construct a theoretical model of economic growth incorporating health capital, to conduct an in-depth analysis of the economic effects of implementing a dynamic BMI reimbursement rate along the saddle path. It will explore the mechanisms through which dynamic reimbursement affects the rate of health capital accumulation, social welfare improvement, and long-term economic growth, and ultimately provide theoretical guidance and international references for the optimization of China’s BMI policy.

In the field of health economics, Grossman (1) studied the impact of economic development on health demand from a microeconomic perspective. Subsequently, Ehrlich and Liu (2) analyzed the mutual influence between health and economic growth from a macroeconomic viewpoint. Since then, a vast body of literature has emerged analyzing the relationship between health and economic growth. These studies are primarily divided into three aspects: changes in productive capacity, shifts in consumer health preferences, and health insurance compensation policies.

Specifically, theoretical and empirical studies have mainly adopted three approaches. First, incorporating health capital into the production function, as Bloom et al. (3) and Barro (4) suggest that health can directly improve labor productivity while reducing health capital depreciation through lower mortality and morbidity rates. Bloom et al. (3) used econometric methods to confirm the positive impact of good health on production efficiency, whereas Barro (4) developed an endogenous growth model that includes health capital to analyze the interaction mechanism between health and economic growth. Second, including health capital or health goods in the utility function, as proposed by Kelly (5) and Atolia et al. (6), who argue that incorporating health status into consumption decisions can achieve the optimal state of health along the entire saddle path. Third, considering the relationship between health status and the duration of illness, as Halliday (7) posits that the accumulation of health capital can reduce the duration of illness and increase labor supply. Halliday (7) embeds health capital into the duration of illness, analyzing the impact of health investments on labor supply and illness duration.

Research on the intrinsic mechanism between health and economic growth has been explored in multiple dimensions both theoretically and empirically. Many theoretical studies have utilized endogenous growth models. For example, Agénor (8) and Barro (4) directly incorporate health capital as a form of human capital into the production function and analyze the relationship between health and economic growth (9).

Empirical studies primarily focus on the impact of health on production efficiency and consumption. For instance, Liu et al. (10) used data from the China Health and Nutrition Survey (CHNS) to investigate the impact of health capital on income productivity among rural residents in China, finding that the income of rural residents is significantly constrained by their health status. Bai and Wu (11) utilized data from the New Cooperative Medical Scheme (NCMS) from the Rural Fixed Observation Point Survey (RFPS) in China and found that the NCMS effectively increased households’ non-medical consumption. Liu (12) using data from the China Health and Nutrition Survey (CHNS) discovered that medical insurance can reduce the excessive supply of family labor, thereby substituting for this self-insurance behavior (13). Moreover, participation in medical insurance can also increase total medical expenditure, reduce out-of-pocket medical expenses (14–16), improve residents’ health status, and lower mortality rates (17). The Pareto improvement from participation in medical insurance can further stabilize household income (12) and enhance social welfare (18).

This paper primarily analyzes the mechanism of action between health and economic growth from a theoretical perspective. Research closely related to this paper includes the studies by Kelly (5) and Atolia et al. (6). Kelly (5) incorporates health capital into the production function within a neoclassical economic growth model to affect changes in total factor productivity, while Atolia et al. (6) incorporate the demand for health goods into the utility function, influencing decisions on health goods consumption. The neoclassical economic growth model can depict the dynamic accumulation paths of material capital and health capital, driving economic growth through the accumulation of material capital and the consumption of medical products. This precisely reflects the characteristics of factor-driven economic development in China over the past 40-plus years. Therefore, within this framework, depicting the mechanism through which material capital gradually accumulates to drive economic growth, how health capital gradually accumulates to affect economic growth, and considering the feedback mechanisms between consumption of medical goods, health capital accumulation, and economic growth, constitutes the primary focus of this paper’s research.

Van Doorslaer et al. (19) found in their study of OECD member countries that about half of the OECD nations exhibit inequities in the utilization of healthcare services, with higher-income groups benefiting more from healthcare services. Furthermore, van Doorslaer et al. (20) and Lu et al. (14), utilizing healthcare insurance data from European, American, and Asian countries, observed similar findings. These studies indicate that, although health insurance effectively reduces income disparities, it also leads to a problem of “reverse redistribution” of wealth (21). Finkelstein et al. (22), using health insurance data from Massachusetts, discovered that adopting progressive compensation policies could increase the insurance coverage rate among low-income populations.

However, the aforementioned studies analyze the impact of basic medical insurance compensation rates on health from a short-term, microeconomic perspective. Clearly, the performance analysis of short-term basic medical insurance cannot represent the changes in residents’ health status in the context of long-term economic growth. Especially considering that China’s economic growth is still on the saddle path toward a steady-state equilibrium, with the level of economic development still far from the steady-state level. Therefore, constructing a neoclassical economic growth theoretical model to solve for the equilibrium solution on the saddle path, and analyzing the interrelationship between health capital accumulation and economic growth under different basic medical insurance compensation policies at various stages of economic development, holds significant theoretical value and practical significance. This also constitutes the second important research content of this paper.

The potential marginal contributions of this paper are as follows: First, by calculating the equilibrium solutions on the saddle path for each period using the Shooting algorithm, it circumvents the error issues associated with linear approximation techniques, obtaining precise solutions at any initial level of material and health capital. Second, this paper constructs an economic growth framework under a factor-driven model to study the relationship between health capital and economic growth. It incorporates the accumulation process of health capital into economic growth, providing a more intuitive explanation of the impact mechanism between health capital and long-term economic growth. This aids in analyzing the long-term and short-term effects of basic medical insurance compensation policies on medical consumption, health capital accumulation, and economic growth. Third, unlike previous discussions that mainly focus on resource allocation at the steady-state equilibrium, this paper discusses dynamic medical cost compensation mechanisms on the path of economic growth, enabling more effective optimization of resource allocation. This approach not only improves residents’ health but also considers the efficiency of economic growth.

The structure of the remaining sections of this paper is as follows: The second section is on methodology. The third section covers results and discussion. In this part, the external effects of dynamic medical insurance compensation rates are studied, as well as the accumulation path of health capital, labor time supply, and economic growth under the influence of a dynamic basic medical insurance compensation system. The final section presents the main conclusions of the paper.

## 2 Methodology

### 2.1 Theoretical model

This paper builds on the Ramsey-Cass-Koopmans model by adding a medical products sector and incorporating health capital into the production function of the final goods sector, thereby constructing a model that includes the production and consumption of medical goods, the accumulation of health capital, and the basic medical insurance system. Unlike most previous studies, this paper solves for the steady-state equilibrium of the theoretical model and then uses the Shooting algorithm to solve for the equilibrium solutions for each period. This approach further explores the dynamic interaction mechanism between the basic medical insurance system and economic growth.

#### 2.1.1 Production sector

To clearly examine the relationship between medical goods producers and general goods producers, we assume that the economic system consists of two types of producers: a medical goods sector and a final goods sector.^1^ We assume that production and consumption occur only between firms and households, implying that all medical goods are exclusively consumed by households. To simplify potential complications arising from technical differences, we assume that there are no frictions in capital flows, such as borrowing or lending, between the two production sectors, and that labor can move freely between the two sectors. Furthermore, we assume that the production of medical goods relies on two inputs: medical capital and labor, and the production function of the medical goods sector is given as follows:


(1) $$h_{t}=B_{t}K_{E,t}^{\varphi }L_{E,t}^{1-\varphi }$$

Where, $h_{t}$ represents the output of medical products in period t , $K_{E,t}$ denotes the physical capital stock in the medical sector in period t , $L_{E,t}$ indicates the labor demand in the medical sector in period t , φ is the output elasticity coefficient of physical capital in the medical products sector, and $B_{t}$ represents the total factor productivity in the medical products sector for period t .

The objective function of the medical products sector, aimed at maximizing profits, is:


$$\pi_{h,t}=P_{t}h_{t}-r_{t}K_{E,t}-W_{t}L_{E,t}$$

Where, $\pi_{h,t}$ represents the profit of the medical products sector in period t , $P_{t}$ is the price of medical products in period t , $r_{t}$ is the marginal cost of capital in the medical products sector in period t , and $W_{t}$ is the wage in the medical products sector in period t .

The first-order conditions for profit maximization in the medical products sector are:


(2) $$r_{t}=\varphi \frac{P_{t}h_{t}}{K_{E,t}}=\varphi P_{t}B_{t}K_{E,t}^{\varphi -1}L_{E,t}^{1-\varphi }$$

(3) $$W_{t}=\left(1-\varphi \right)\frac{P_{t}h_{t}}{L_{E,t}}=\varphi P_{t}B_{t}K_{E,t}^{\varphi }L_{E,t}^{-\varphi }$$

In fact, several studies (3, 23) have confirmed that health status can affect the total factor productivity (TFP) of the production sector. Accordingly, we assume that health capital influences total factor productivity. We also continue to assume that the production of medical goods depends on medical capital and labor. Therefore, following Kelly (5), the representative final goods sector has the following production function:


(4) $$Y_{t}=\left(A_{t}H_{t}^{\gamma }\right)K_{F,t}^{\alpha }L_{F,t}^{1-\alpha }$$

Where, $Y_{t}$ represents the output of the final goods sector in period t , $H_{t}$ denotes the stock of health capital in period t , $A_{t}H_{t}^{\gamma }$ signifies the total factor productivity in the final goods sector in period t , $K_{F,t}$ is the physical capital stock in the final goods sector in period t , $L_{F,t}$ indicates the labor demand in period t , α is the output elasticity coefficient of physical capital in the final goods sector, and γ represents the elasticity coefficient of health capital in the final goods sector.

The objective function aimed at maximizing profits for the final goods sector is:


$$\pi_{t}=Y_{t}-r_{t}K_{F,t}-W_{t}L_{F,t}$$

Where, $\pi_{t}$ represents the profit of the final goods sector in period t , $r_{t}$ is the marginal cost of capital in the final goods sector in period t , and $W_{t}$ is the wage in the final goods sector in period t .

The first-order conditions for profit maximization in the final goods sector are:


(5) $$r_{t}=\alpha \frac{Y_{t}}{K_{F,t}}=\alpha \left(A_{t}H_{t}^{\gamma }\right)K_{F,t}^{\alpha -1}L_{F,t}^{1-\alpha }$$

(6) $$W_{t}=\left(1-\alpha \right)\frac{Y_{t}}{L_{F,t}}=\left(1-\alpha \right)\left(A_{t}H_{t}^{\gamma }\right)K_{F,t}^{\alpha }L_{F,t}^{-\alpha }$$

#### 2.1.2 Household sector

In this paper, it is assumed that each household in the economic system aims to maximize lifetime utility, with the population growth rate of the household being zero. The discount rate for time preference is denoted by β , χ represents the elasticity of substitution for leisure within the same period, ω signifies the elasticity of substitution for the demand for medical products within the same period, δ is the depreciation rate of physical capital, and $\delta_{h}$ is the depreciation rate of health capital.

The utility maximization function for the household sector is:


$$\max\left[\overset{\infty }{\underset{t=0}{\sum }}\beta^{t}U\left(C_{t},l_{t},h_{t}\right)\right]$$

This paper, drawing on the design approach of the utility function by Herrendorf et al. (24) and Atolia et al. (6), and incorporating the empirical research findings of Finkelstein et al. (25), assumes the utility function for the representative household as follows:^2^


$$U\left(C_{t},l_{t},h_{t}\right)=\ln\left(C_{t}-\bar{C}\right)+\chi \ln l_{t}+\omega \ln h_{t}$$

Where, $\bar{C}$ represents basic food consumption.

The budget constraint equation for the representative household shown as in Equation 7:


(7) $$C_{t}+I_{t}+\left(1-s_{t}\right)P_{t}h_{t}+\Pi_{\;t}\leq r_{t}K_{t}+W_{t}L_{t}-T_{t}$$

Where, $C_{t}$ denotes the household’s consumption expenditure in period t , $I_{t}$ represents the household’s investment expenditure in physical capital in period t , and the stock of physical capital $K_{t}$ is the sum of physical capital in the medical products sector $K_{E,t}$ and the final products sector $K_{F,t}$ , i.e., $K_{t}=K_{E,t}+K_{F,t}$ . The labor supply $L_{t}$ is the sum of labor supply to the medical products sector $L_{E,t}$ and the final products sector $L_{F,t}$ , i.e., $L_{t}=L_{E,t}+L_{F,t}$ . $h_{t}$ represents the demand for medical products, $\left(1-s_{t}\right)P_{t}h_{t}$ denotes the household’s out-of-pocket medical consumption expenditure in period t , $s_{t}$ is the rate of basic medical insurance compensation in period t , $T_{t}$ signifies the household’s lump-sum tax in period t , and $\Pi_{\;t}$ is the premium paid for basic medical insurance in period t .

The dynamic accumulation equation for physical capital is typically represented as follows:


(8) $$K_{t+1}=\left(1-\delta \right)K_{t}+I_{t}$$

By optimizing the utility function, the intertemporal Euler equation for consumption can be derived as follows:


(9) $$\left(C_{t+1}-\bar{C}\right)=\left(C_{t}-\bar{C}\right)\beta \left(r_{t+1}+1-\delta \right)$$

The contemporaneous substitution equation for consumption and leisure can be expressed as follows:


(10) $$\chi \left(C_{t}-\bar{C}\right)=W_{t}l_{t}$$

The contemporaneous substitution equation between non-medical consumption and medical consumption expenditure can be formulated as follows:


(11) $$\omega \left(C_{t}-\bar{C}\right)=\left(1-s_{t}\right)P_{t}h_{t}$$

The paper posits that health status depreciates due to factors such as age and illness, but because household health investment decisions are influenced by both consumption and investment motives, they continually purchase medical products in the medical product market to improve health status (7). It is assumed that medical products are essential goods, and a certain level of medical product supply is necessary to facilitate the accumulation of health capital. The minimum consumption of medical products required to promote health capital accumulation is denoted as $\hat{h}$ . In the early stages of economic development, the supply of medical products is limited, falling below $\hat{h}$ . As the economy expands, the household sector’s demand for medical products continuously increases, and when $h_{t}>\hat{h}$ , the investment in health capital gradually leads to its accumulation. Therefore, following Huang and Huffman (26), the dynamic accumulation equation for health capital can be expressed as:


(12) $$H_{t+1}=\left(1-\delta_{h}\right)H_{t}+I_{H,t}$$

Where, $I_{H,t}$ represents the investment in health capital in period t , and when $h_{t}>\hat{h}$ , $I_{H,t}=h_{t}-\hat{h}$ , where $\delta_{h}$ denotes the depreciation rate of health capital. When the consumption of medical products $h_{t}\leq \hat{h}$ , $I_{H,t}=0$ , meaning that it only suffices for normal productive activities and is insufficient to promote positive accumulation of health capital $H_{t}$ .

#### 2.1.3 Household sector

In this paper, it is assumed that the revenue of the medical insurance sector comes from government fiscal subsidies and premiums paid by individuals, and the expenditures of the medical insurance sector are limited to reimbursements for basic medical insurance costs. Consequently, the budget constraint equation for the medical insurance sector is as Equation 13:


(13) $$T_{t}+\Pi_{\;t}=s_{t}P_{t}h_{t}$$

Where, $T_{t}$ represents the government fiscal subsidies, and $s_{t}$ denotes the compensation ratio of the medical insurance premium paid by the household sector.

When the product market in the economic system reaches equilibrium (i.e., the market clears), the following equation is satisfied:


(14) $$Y_{t}+P_{t}h_{t}=C_{t}+I_{t}+P_{t}h_{t}$$

When the labor market in the economic system reaches equilibrium (i.e., the market clears), the following equation is satisfied:


(15) $$L_{t}+l=1$$

## 3 Results and analysis

### 3.1 Parameter calibration

During the process of China’s economic growth, the short-term fluctuations of various economic parameters remain quite small. In the neoclassical economics framework, it is common practice to use real-world data to estimate fixed parameters for effectively analyzing economic growth paths. However, the main focus of this paper is not on parameter estimation itself; previous studies have already provided relatively reasonable estimates. Therefore, taking into account the Chinese economic context, we adopt appropriate parameters based on existing research.

Fan and Zhang (27), using the perpetual inventory method to measure China’s capital depreciation series over the past 15 years, found that the depreciation rate of physical capital is δ=0.096 . Drawing on employment figures for the national industrial sector, they estimated a capital-output elasticity of α=0.51 for the final goods sector. Consistent with their findings, this paper sets the depreciation rate of physical capital to δ=0.096 and the capital-output elasticity to α=0.51 . We typically consider the final goods sector as the baseline sector and set its total factor productivity (TFP) to $A_{t}=1$ . Referring to Atolia et al. (6), we set the capital elasticity coefficient in the medical goods sector to φ=0.55 , its TFP to $B_{t}=0.99$ , and the substitution elasticity for medical goods demand to ω=0.15 . Under these parameter settings, the share of output from the medical goods sector in the overall economy aligns reasonably well with reality. Drawing on Kelly (5), who estimated the depreciation rate of health capital and the elasticity of health capital in the final goods sector, we select $\delta_{h}=0.04$ and γ=0.05 .

Moreover, following Fan and Zhang (27) in calculating the annual utility discount factor for China, we set β=0.975 . Based on the neoclassical economic model proposed by Zhou and Fan (28) that incorporates mechanisms for reducing carbon emission intensity, we set the elasticity of utility substitution for leisure to χ=0.2 . Regardless of other types of consumption, individuals must at least meet their basic subsistence consumption. According to the methodology of Leukhina and Turnovsky (29), we set the baseline food consumption to $\bar{C}=0.1468$ . By calibrating the model’s parameters to maximize the model’s fit, we set the threshold level of medical goods consumption to $\hat{h}=0.211$ .

To present these parameters clearly, Table 1 summarizes the parameters, symbols, and their values.

**Table 1:** Parameters, symbols, and values.

Parameters	Symbols	Values
Physical capital depreciation rate	δ	0.096
Capital elasticity in the final goods sector	α	0.51
TFP in the Final Goods Sector	$A_{t}$	1
The elasticity of health capital in the final goods sector	γ	0.05
The capital elasticity for the medical products sector	φ	0.55
TFP in the medical products sector	$B_{t}$	0.99
The elasticity of substitution for medical goods demand	ω	0.15
The depreciation rate of health capital	$\delta_{h}$	0.04
The annual discount rate	β	0.975
The elasticity of substitution for leisure	χ	0.2
The food consumption	$\bar{C}$	0.1468
The threshold for medical product consumption	$\hat{h}$	0.211

These calibrated parameters are designed to refine the model’s accuracy and relevance to the specific economic and health dynamics of China. By adjusting these parameters, the paper aims to closely mirror real-world conditions and provide insights into the impacts of economic policies and health capital on China’s growth trajectory.

To facilitate readers’ understanding of the economic terminology used in the model, we provide detailed explanations in Table 2.

**Table 2:** Economic interpretation of variables and parameters.

Name	Symbol	Economic interpretation
Physical capital	$K_{t}$	As firms continue to invest, capital accumulates as a stock of tangible assets, which typically depreciates and deteriorates over time. In this paper, it is defined as the sum of capital stock in the final goods sector and the medical goods sector.
Investment	$I_{t}$	In order to meet operational needs, firms may increase or decrease capital through investment activities, which is a flow variable. It includes investment in both the final goods sector and the medical goods sector.
Leisure time	$l_{t}$	It represents the amount of time workers allocate to rest, in contrast to working hours. In this paper, the total available time is normalized to 1, so both labor and leisure time lie within the interval [0, 1]
Medical goods consumption	$h_{t}$	Assuming that the medical goods produced by firms are entirely consumed by households, it represents the quantity of medical goods.
Output of the final goods sector	$Y_{t}$	By normalizing the price of the final goods sector to 1, the resulting value corresponds to the total output of the final goods sector.
Health capital	$H_{t}$	A stock of health capital that can be increased through investment in medical consumption.
Capital elasticity	α,φ,γ	If the entire economy is viewed as a factory, then “capital” can be understood as machinery, equipment, and buildings. A higher capital elasticity coefficient implies that an increase in capital input leads to a more substantial rise in output; conversely, a lower elasticity indicates that additional capital has a relatively limited effect on output.
Capital depreciation rate	$\delta ,\delta_{h}$	The capital depreciation rate can be understood as the speed at which machinery and equipment wear out or become obsolete over time. Just like household appliances that deteriorate with use and eventually require repair or replacement, a higher depreciation rate means that capital assets such as machines and buildings degrade more quickly each year, thus requiring greater investment to offset this loss.
Discount rate	β	It reflects the degree to which individuals prefer the present over the future. A higher utility discount rate indicates a stronger preference for immediate gratification rather than delaying consumption for future returns. For example, when holding a sum of money, a person with a high discount rate is more likely to spend or consume it right away, placing less value on benefits that arrive years later; in contrast, someone with a low discount rate is more willing to wait for future gains.
Elasticity of substitution for leisure	χ	It primarily measures the degree of substitutability between work and leisure. In other words, if the satisfaction individuals derive from leisure time can be easily replaced by money or consumption, then the elasticity is high. Conversely, if individuals have a strong preference for leisure and are unwilling to give up rest in exchange for more work, the elasticity is low. It reflects how much leisure individuals are willing to sacrifice for additional income—or vice versa—while keeping overall utility constant.

### 3.2 Solving for the saddle path

The Shooting algorithm employed in this paper to calculate the equilibrium solutions on the saddle path for each period works as follows, starting from given initial values of physical and health capital in the first period and progressively solving for the equilibrium solutions on the saddle path for each subsequent period:

To verify the convergence of the saddle path calculated by the Shooting algorithm to the steady-state values, and to compare these with the actual calculated steady-state values, the `fsolve` function from MATLAB’s Optimization Toolbox is used to solve for the steady-state values of the various variables.

Second, set the maximum number of periods for the calculation, for example, T=1000 , to ensure that the economic system converges to the steady state by t≤T . Simultaneously, specify the initial levels of physical capital $K_{1}$ and health capital $H_{1}$ , along with a sequence of basic medical insurance compensation rates $\left\{s_{t}\right\}$ . Define a wide capital interval for the physical capital of the second period, $K_{2}$ , as $\left[\underline{K},\bar{K}\right]$ , where $\underline{K}$ and $\bar{K}$ respectively represent the lower and upper bounds of the capital for the second period. Here, $\underline{K}$ could be set to $K_{1}$ , and $\bar{K}$ to the capital steady-state value solved by `fsolve`. Using the idea of binary search, assume $K_{2}=\left(\underline{K}+\bar{K}\right)/2$ , and based on $K_{1}$ , $K_{2}$ , and Equations 1–6, 10, 11, 14, 15, calculate $I_{1}$ , $L_{1}$ , $l_{1}$ , $h_{1}$ , $Y_{1}$ , $C_{1}$ , $r_{1}$ , $W_{1}$ , $K_{E,1}$ , $K_{F,1}$ , $L_{E,1}$ and $L_{F,1}$ . Calculate $H_{2}$ based on $H_{1}$ , $h_{1}$ , and Equation 12. Calculate $I_{2}$ , $L_{2}$ , $l_{2}$ , $h_{2}$ , $Y_{2}$ , $C_{2}$ , $r_{2}$ , $W_{2}$ , $K_{E,2}$ , $K_{F,2}$ , $L_{E,2}$ and $L_{F,2}$ using Equations 1–6, 9–11, 14, 15. Then, calculate $K_{3}$ and $H_{3}$ using Equations 8, 12. Repeat these steps until reaching period T .

Assume $K_{2}^{*}$ represents the true value of the physical capital in the second period. If $K_{2}>K_{2}^{*}$ , this would lead to excessive capital accumulation on the saddle path, resulting in $C_{t}<0$ . In this case, adjust $\bar{K}$ by setting $\bar{K}=K_{2}$ . Conversely, if $K_{2}<K_{2}^{*}$ , this would lead to excessive consumption on the saddle path, causing insufficient accumulation of physical capital, i.e., $K_{t}<0$ . Therefore, adjust $\underline{K}$ by setting $\underline{K}=K_{2}$ . Repeat the calculation of the saddle path until $|\bar{K}-\underline{K}|\leq 10^{-12}$ converges. The calculation stops when convergence is achieved, resulting in the equilibrium solutions for each period along the saddle path.

Finally, compare the steady-state values converged upon by the Shooting algorithm with the steady-state values solved by the `fsolve` function. This comparison determines the effectiveness of the Shooting algorithm in achieving steady-state convergence.

### 3.3 Analysis of the external effects of dynamic basic medical insurance compensation

Accumulated health capital is considered a public good for businesses, making it difficult to incorporate it into the cost of production. Individuals’ investment in health is limited by budget constraints, leading to insufficient health investments. Therefore, by establishing a basic medical insurance compensation system to promote medical product consumption, which in turn encourages the accumulation of health capital and improves social welfare, the government demonstrates the positive externality of basic medical insurance compensation.

#### 3.3.1 The setting of three types of basic medical insurance compensation rates

In the real economy, health capital accumulates along the saddle path as economic development progresses, and the economy remains on the growth path when the government establishes a medical insurance system. Therefore, considering the medical insurance compensation rate solved at the theoretical model’s steady state as the globally optimal rate may lead to a problem of too low a compensation rate on the saddle path. Particularly in the early stages of economic development, when there is insufficient accumulation of health capital, merely implementing the steady-state basic medical insurance compensation rate cannot rapidly accumulate health capital. This may cause consumption to reach its steady state level sooner, resulting in certain welfare losses, hence referred to as the steady-state medical insurance compensation rate. Without establishing a medical insurance system, i.e., no medical insurance compensation, not only would the accumulation rate of health capital be too slow, but the health capital would also end up at a lower level. Consequently, consumption, output, and other economic indicators would also be lower, hence referred to as the no medical insurance compensation rate.

Hence, we posit that the basic medical insurance compensation rate should decrease dynamically over time. Given the non-linear nature of a saddle-path growth trajectory, we argue that the decrease in the basic medical insurance compensation rate should also be non-linear. Drawing on Fan et al. (30), who used a single-variable quadratic function to depict a dynamic environmental tax and considering the typical “rise-then-fall” property of a quadratic function, we employ only the declining segment of such a curve. This is why we choose a one-dimensional quadratic function.

**Figure 1:**
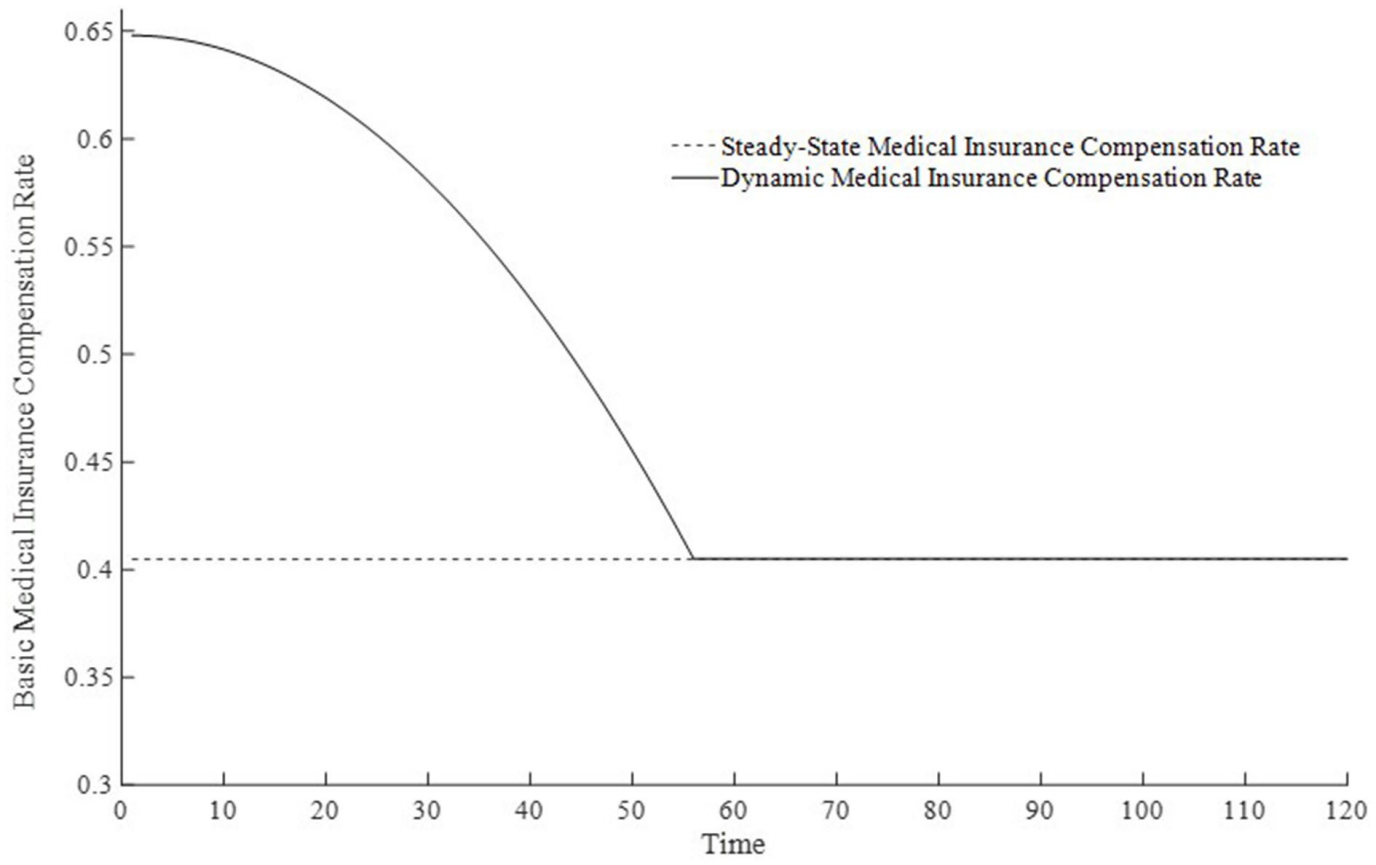
Changes in basic medical insurance compensation rates at different times.

Based on the above analysis, considering the differences in the implementation strength of the basic medical insurance system, this paper sets three types of basic medical insurance compensation rates: no basic medical compensation rate, steady-state basic medical compensation rate, and moderate basic medical compensation rate. Here, no medical compensation means the medical insurance expense compensation rate is 0; the steady-state compensation rate is the optimal compensation rate obtained through steady-state equilibrium solution; the moderate medical compensation rate is higher than the steady-state medical insurance compensation rate^3^. To promote the rapid accumulation of health capital, this paper sets the basic medical insurance compensation rate to be progressively decreasing, that is, a moderate medical insurance compensation rate, which is clearly also a dynamic medical insurance compensation rate. Figure 1 displays the changes in medical compensation rates over time, namely the curve of changes in the steady-state medical compensation rate and the moderate medical insurance compensation rate.

#### 3.3.2 From the perspective of dynamic medical insurance compensation based on changes in consumption, out-of-pocket medical expenses, and disposable income

**Figure 2:**
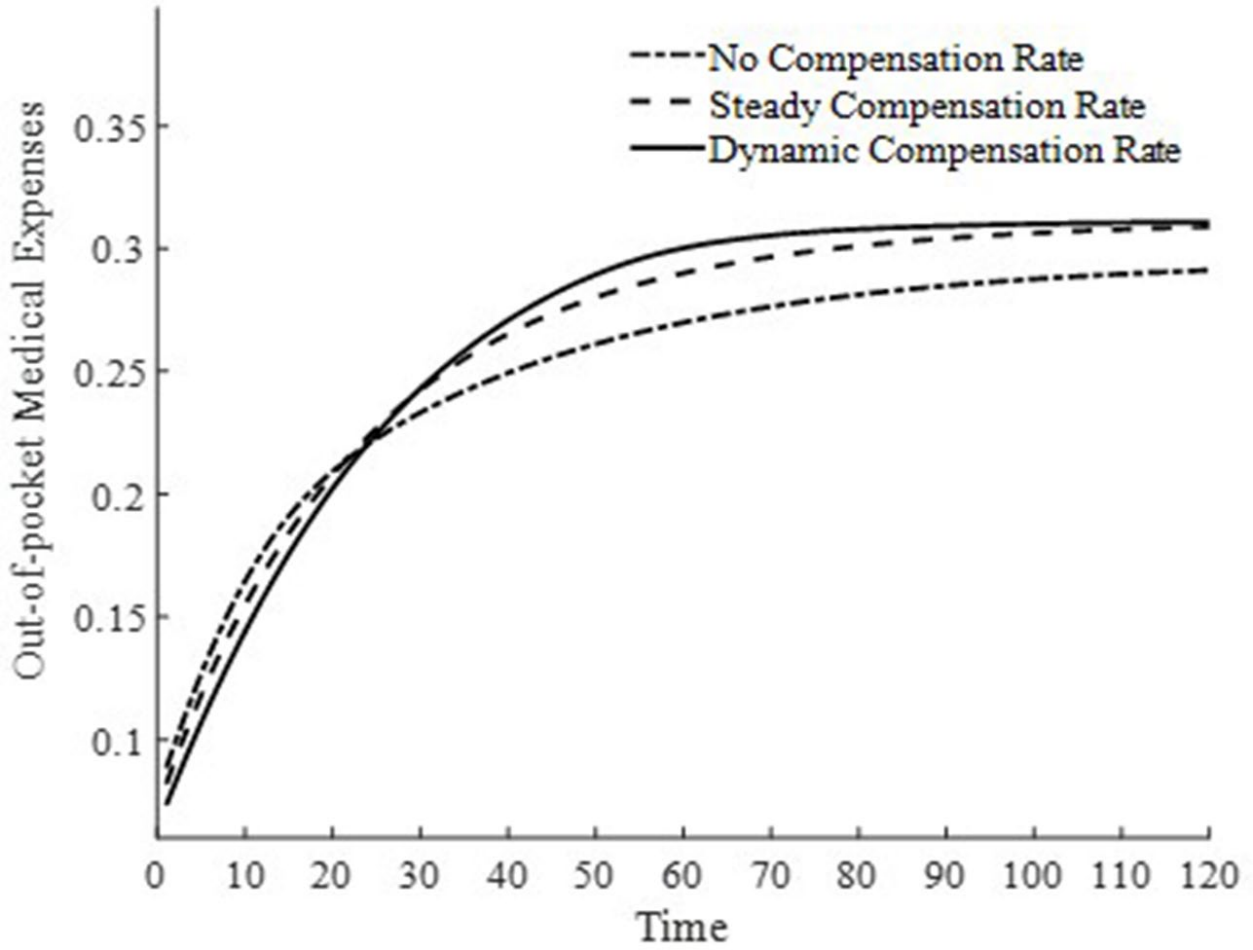
Level of non-medical consumption.

This paper presents in Figure 2 the trend of changes in equilibrium non-medical consumption along the saddle path under three different medical insurance compensation rates. Consumption, a key variable in the utility function, serves as an indicator of welfare levels. From a steady-state perspective, regardless of the presence of a basic medical insurance compensation rate, it takes approximately 120 years to reach the steady-state level of non-medical consumption. Comparatively, the presence of a basic medical insurance compensation rate achieves a higher level of non-medical consumption, indicating that the basic medical insurance policy plays a significant role. The medical insurance system can promote the consumption of medical goods, thereby rapidly accumulating health capital, improving health conditions, enhancing production efficiency, and achieving higher welfare levels.

When two basic medical insurance compensation policies achieve the same steady-state level of non-medical consumption, the non-medical consumption under a dynamic basic medical insurance compensation rate reaches its steady state about 30 years earlier than that under a steady-state basic medical insurance compensation rate. This is because the differences in the basic medical insurance compensation rates on the saddle path only affect the trajectory on the saddle path. This indicates that, with other influencing factors remaining constant, the difference in basic medical insurance compensation rates along the economic growth path does not affect the steady-state level of non-medical consumption. Therefore, welfare analysis based on the level of non-medical consumption along the economic growth path demonstrates that a dynamic basic medical insurance compensation rate is optimal.

**Figure 3:**
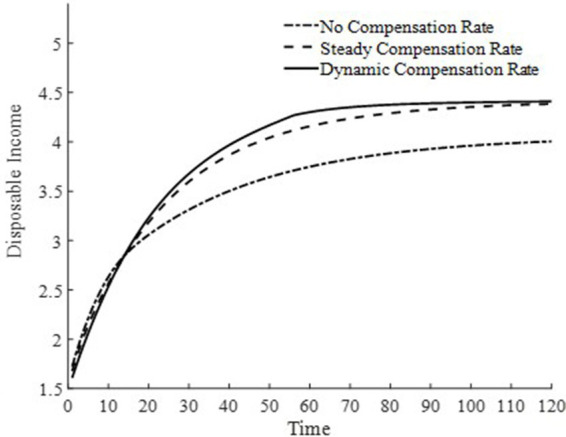
Level of medical consumption.

**Figure 4:**
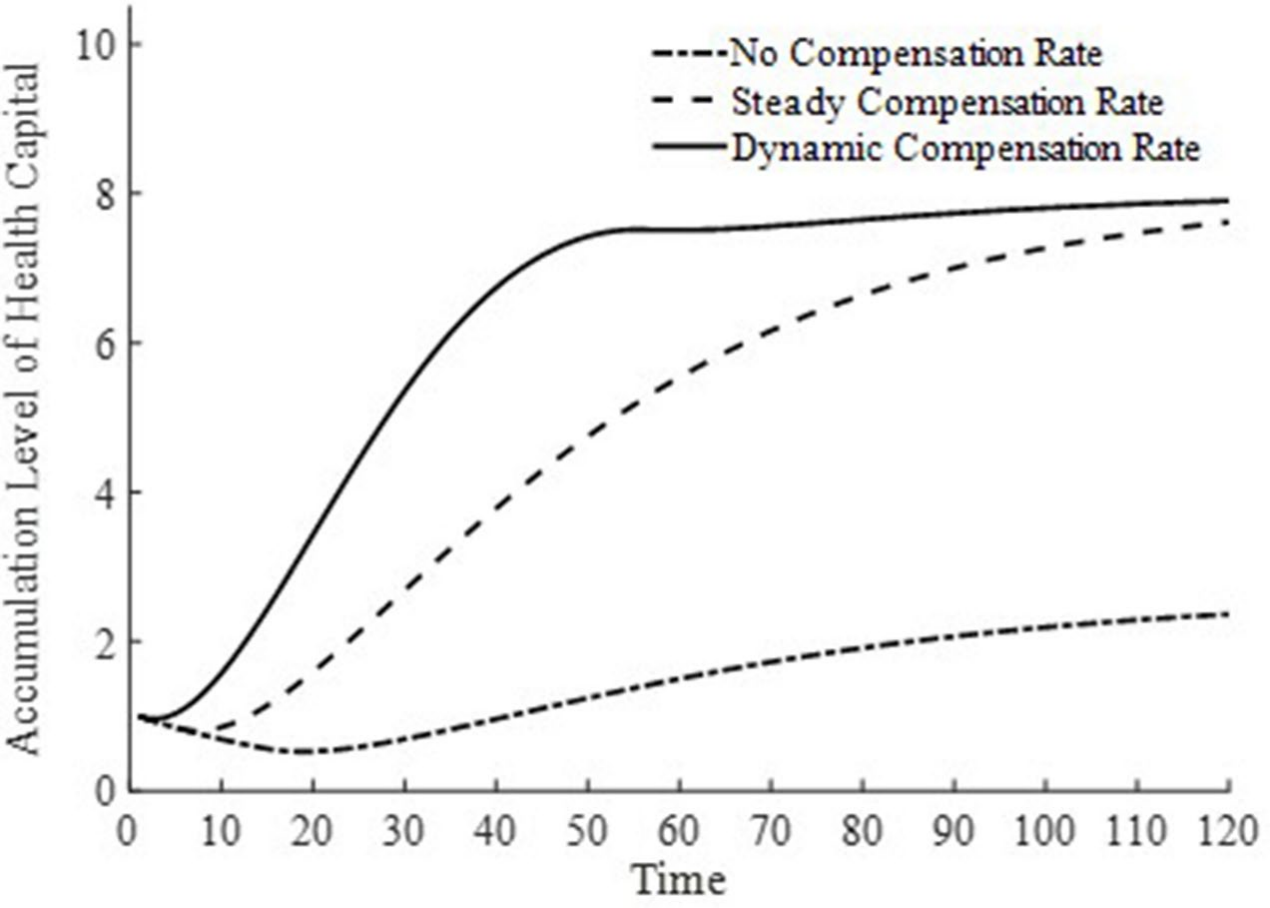
Out-of-pocket medical expenses.

**Figure 5:**
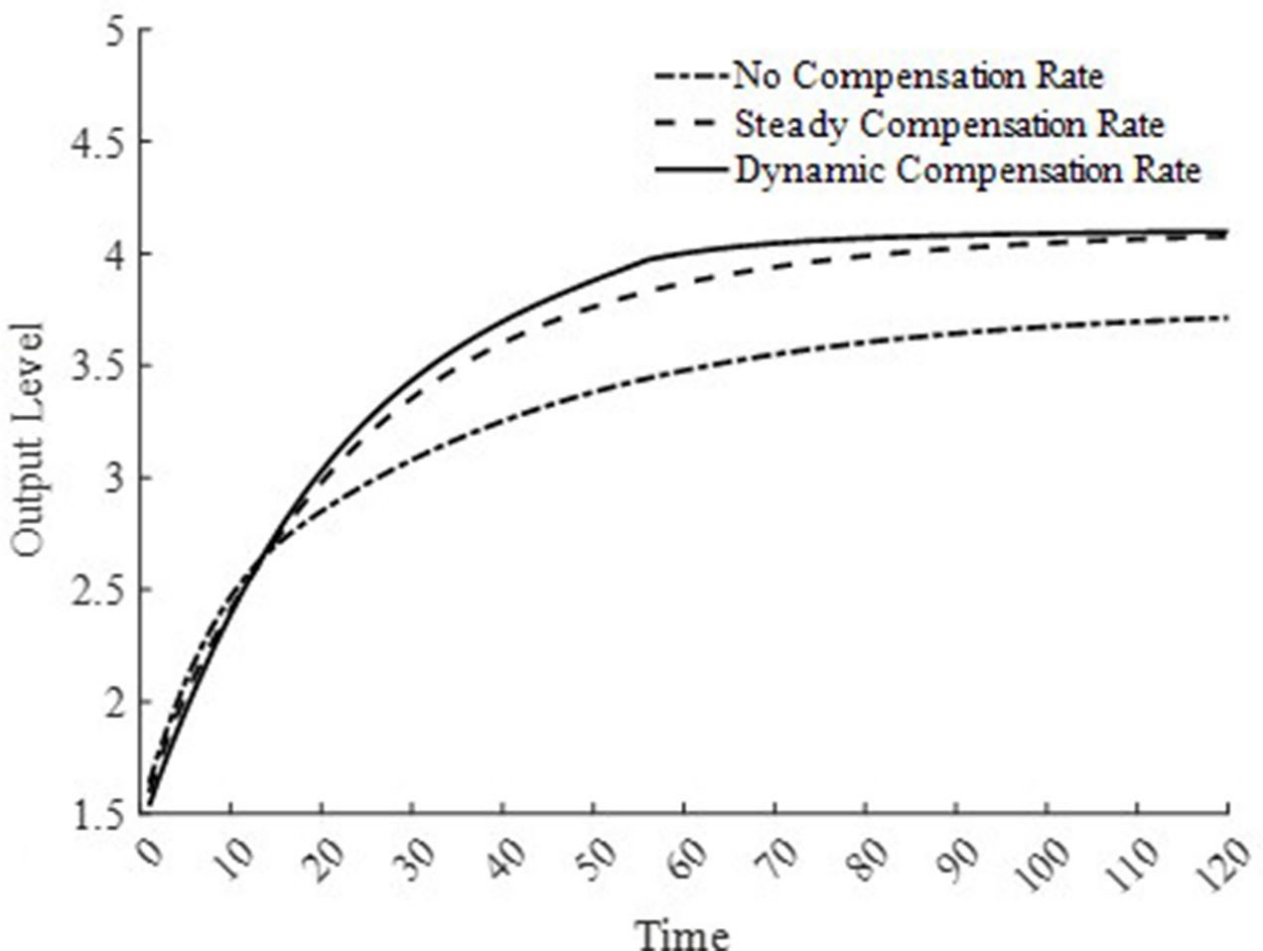
Disposable income.

To further delve into the externalities of dynamic basic medical insurance compensation rates, Figures 3–5 present the differences in equilibrium medical goods consumption, out-of-pocket medical expenses, and disposable income levels under three types of basic medical insurance compensation rates. Figures 3–5 demonstrate that without a medical insurance system, medical consumption struggles to drive health capital accumulation to a higher level, thereby failing to bring about higher levels of disposable income. Clearly, this lower demand for medical products is insufficient. With a basic medical insurance compensation system in place, both the consumption of medical products and out-of-pocket medical expenses are at a higher level when in steady state, as indicated by Figure 1 and Equation 11. This suggests that higher non-medical consumption matches greater medical consumption capacity, where medical consumption can improve health status and thus achieve higher levels of disposable income. At this point, after establishing a basic medical insurance system, the basic medical insurance plays a leveraging role, promoting an overall increase in the society’s consumption level.

Although a steady-state basic medical insurance compensation rate can also promote medical consumption, comparatively, a dynamic basic medical insurance compensation rate results in a higher level of medical product consumption throughout the entire economic growth path. Clearly, in the early stages of economic development, a higher basic medical insurance compensation rate significantly promotes the consumption of medical goods. Moreover, due to the sufficient compensation strength of medical insurance in the early stages of economic development, the out-of-pocket medical expenses for households are relatively low during this phase. This is beneficial for alleviating the problem of households being unable to afford higher medical expenses due to lower disposable income at this stage. Therefore, a dynamic basic medical insurance compensation rate, by promoting the consumption of medical goods, drives economic growth and achieves a win-win result of increasing medical product demand and continuously improving the level of non-medical consumption.

### 3.4 Accumulation path of health capital, labor time supply, and economic growth under a dynamic medical insurance compensation system

Building on the analysis of external effects under the influence of a dynamic basic medical insurance compensation system, this paper will further analyze the role of dynamic medical insurance compensation rates in health capital accumulation, long-term labor supply, and economic growth along the saddle path.

#### 3.4.1 The relationship between health capital accumulation and economic growth under a dynamic basic medical insurance compensation system

**Figure 6:**
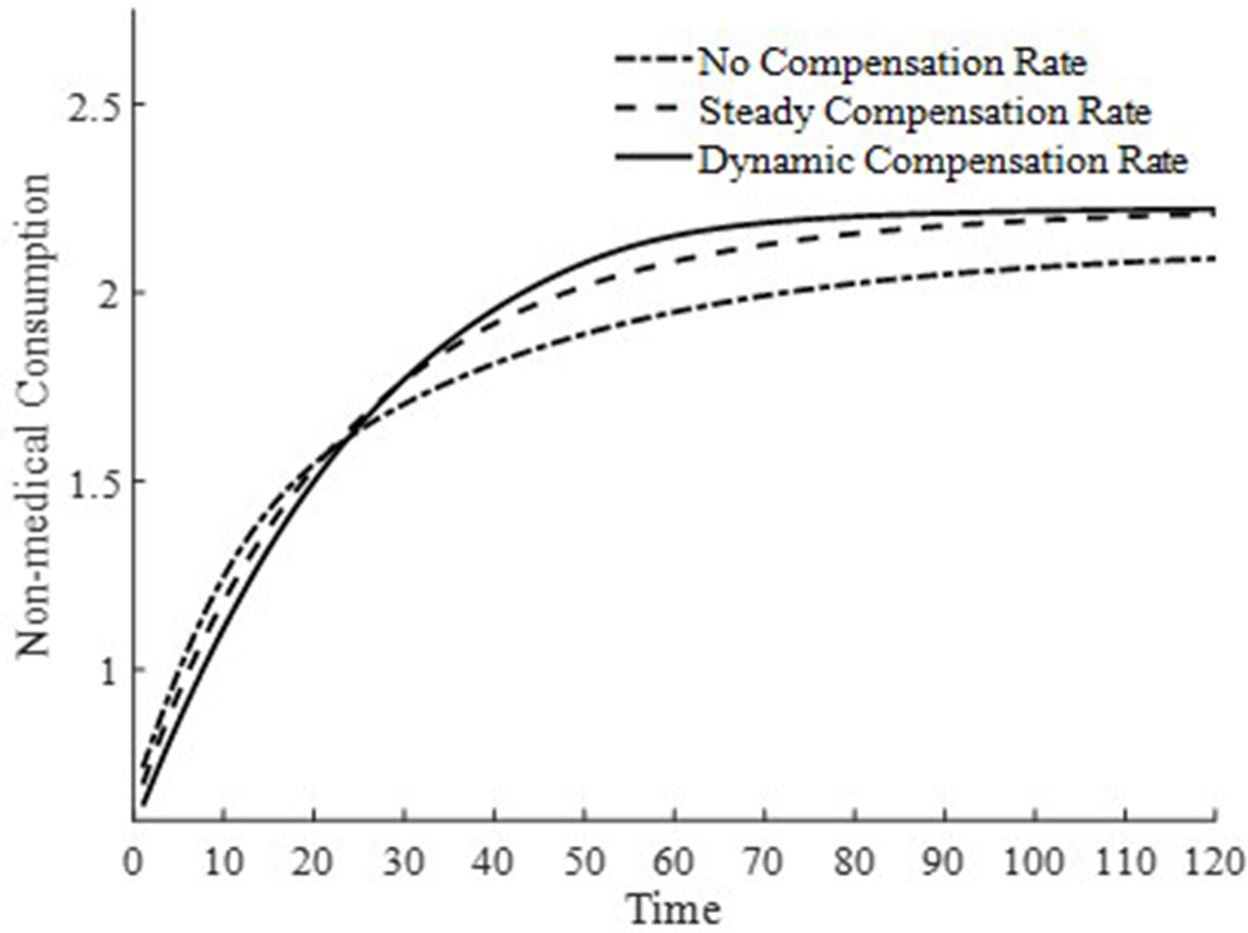
Health capital accumulation level.

**Figure 7:**
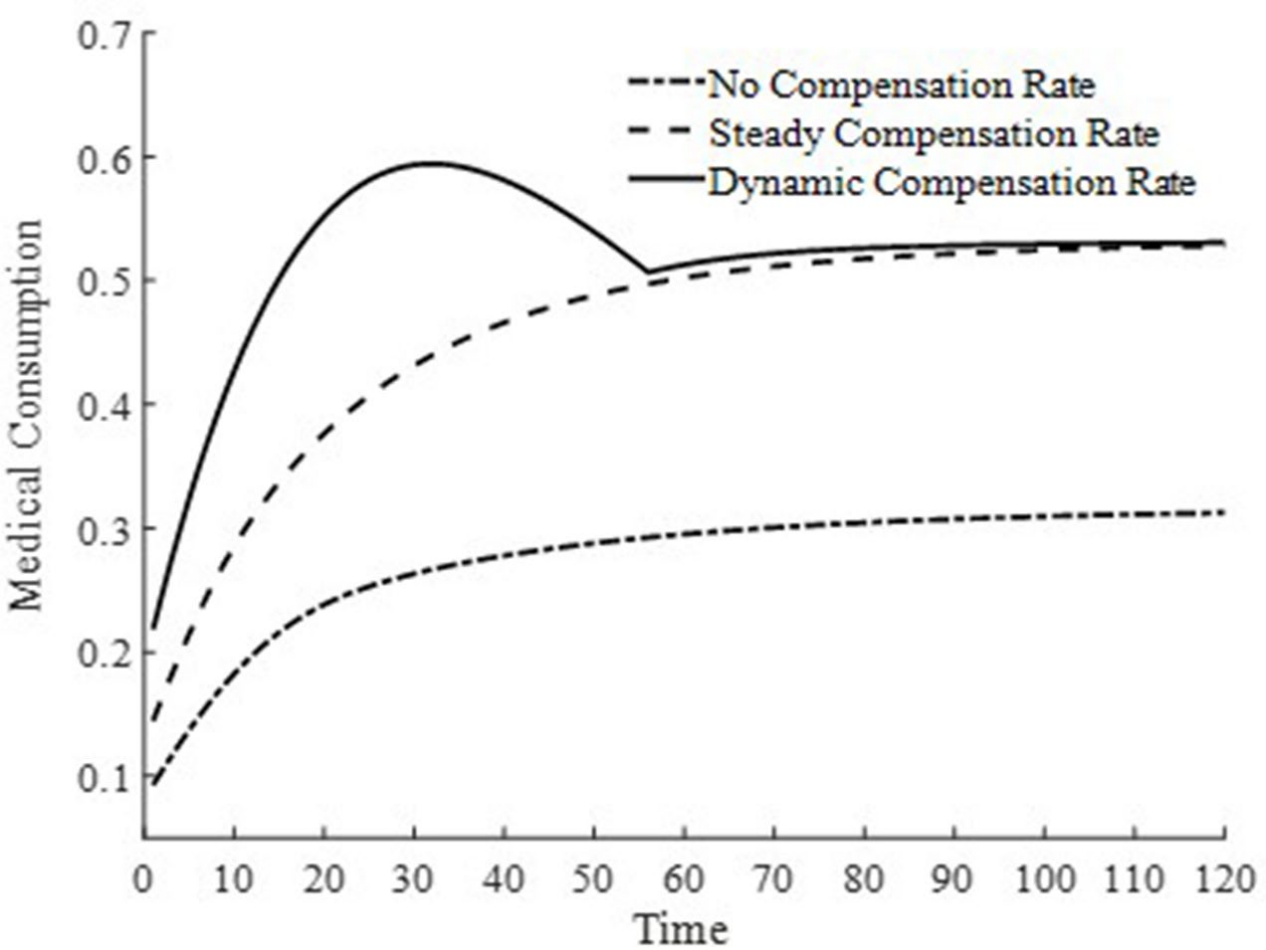
Output level.

In Figures 6, 7, this paper illustrates the saddle path of health capital accumulation and the equilibrium output level under three basic medical insurance compensation rates. Clearly, both health capital and equilibrium output levels are at a lower level under a scenario without medical insurance compensation. Combined with Figure 1 and Equation 11, the lower equilibrium output level decreases the level of non-medical consumption as well as medical product consumption, and inhibits the accumulation of health capital. At the same time, the lower accumulation of health capital also results in lower production efficiency for businesses, leading to lower equilibrium output levels. The level of health capital and equilibrium output level mutually influence each other.

In contrast, under both the steady-state and dynamic basic medical insurance compensation rates, there are higher levels of health capital and equilibrium output. However, as observed from Figures 6, 7, under a dynamic basic medical insurance compensation rate on the saddle path, a higher compensation rate can more rapidly increase medical consumption, thereby accelerating the process of health capital accumulation. The rapid accumulation of health capital further promotes the equilibrium output to reach its steady-state level sooner. Therefore, along the entire saddle path, a dynamic basic medical insurance compensation rate can achieve higher levels of health capital and equilibrium output.

#### 3.4.2 The relationship between labor time supply and economic growth under a dynamic basic medical insurance compensation system

**Figure 8:**
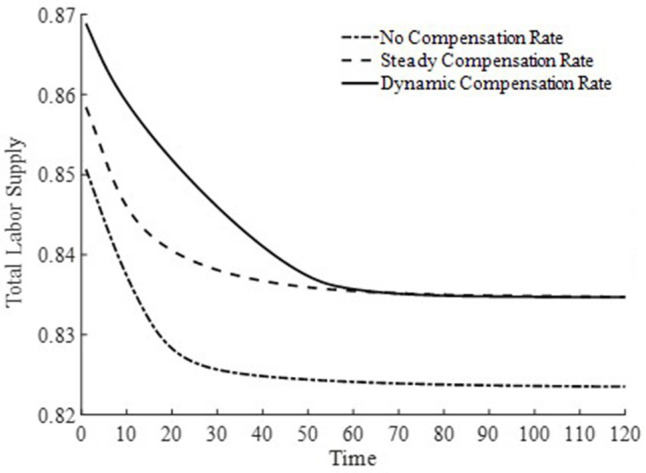
Total labor supply.

**Figure 9:**
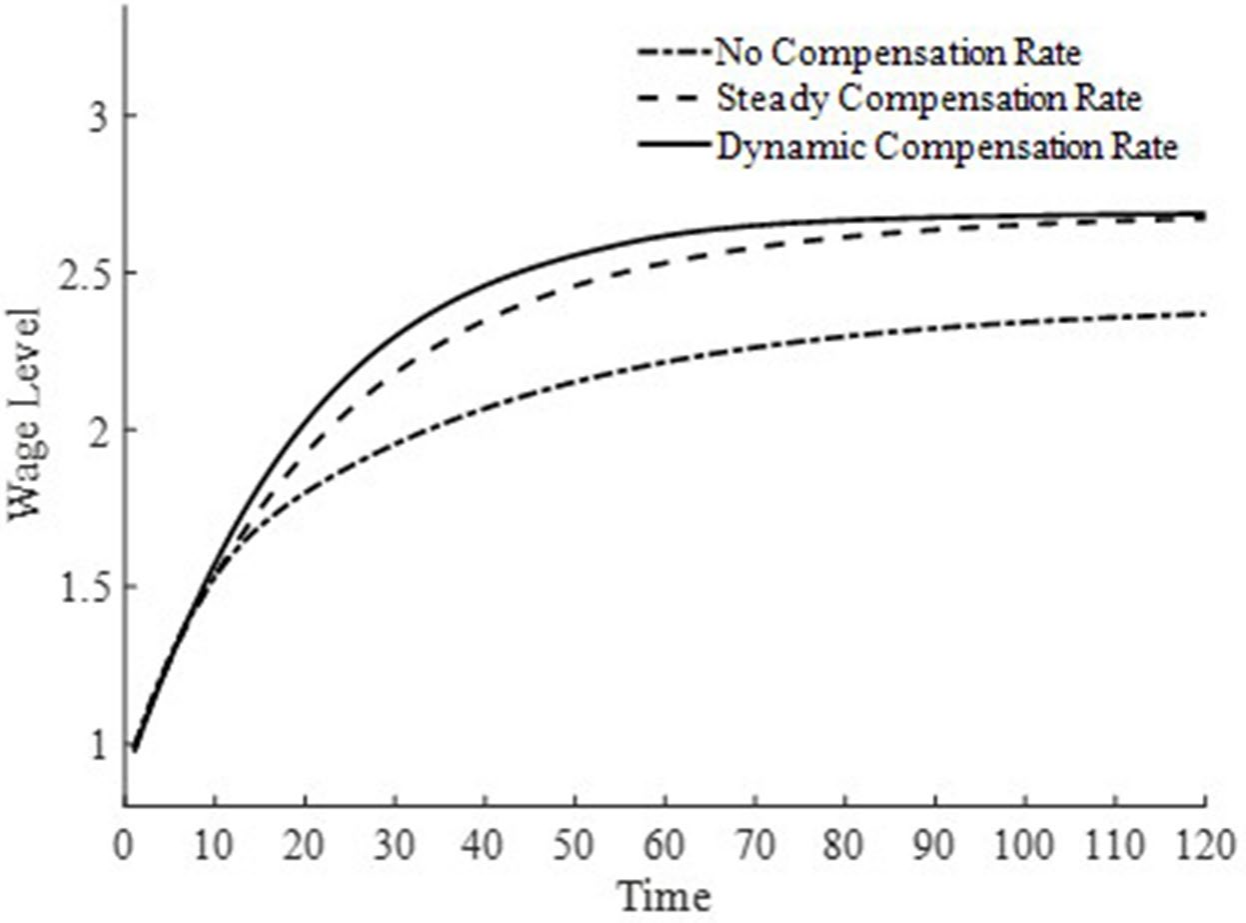
Wage levels.

Figures 8, 9 depict the changes in the saddle path for the total labor time supply and equilibrium wage levels under three types of basic medical insurance compensation rates. Compared to steady-state and dynamic basic medical insurance compensation rates, the equilibrium wage level under no basic medical insurance compensation rate is lower. This is because, in the two sectors where labor is fully mobile, the level of medical product consumption is lower without basic medical insurance compensation. Combined with Figure 6, the production efficiency in the final goods sector is relatively lower, and both sectors together result in lower wage levels. Consequently, the lower wage levels lead to a lower supply of labor time.

Under a dynamic basic medical insurance compensation rate along the saddle path, higher wage levels are achieved, along with a greater supply of labor. Compared to the steady-state basic medical insurance compensation rate, the dynamic compensation rate on the saddle path is higher, leading to higher levels of medical consumption and a more rapid process of health capital accumulation. This, in turn, increases the demand for labor in the medical production sector and allows wages to reach higher levels more quickly. The steady-state basic medical insurance compensation rate, on the other hand, appears to have an issue of insufficient compensation on the saddle path, making it difficult for wage levels to grow rapidly. Therefore, a dynamic basic medical insurance compensation rate not only encourages households to provide more labor supply but also enables households to receive higher labor remuneration. Clearly, this brings about an “income enhancement effect” on the improvement of residents’ health status (23).

#### 3.4.3 Under the dynamic basic medical insurance compensation system: changes in labor time supply among different sectors

**Figure 10:**
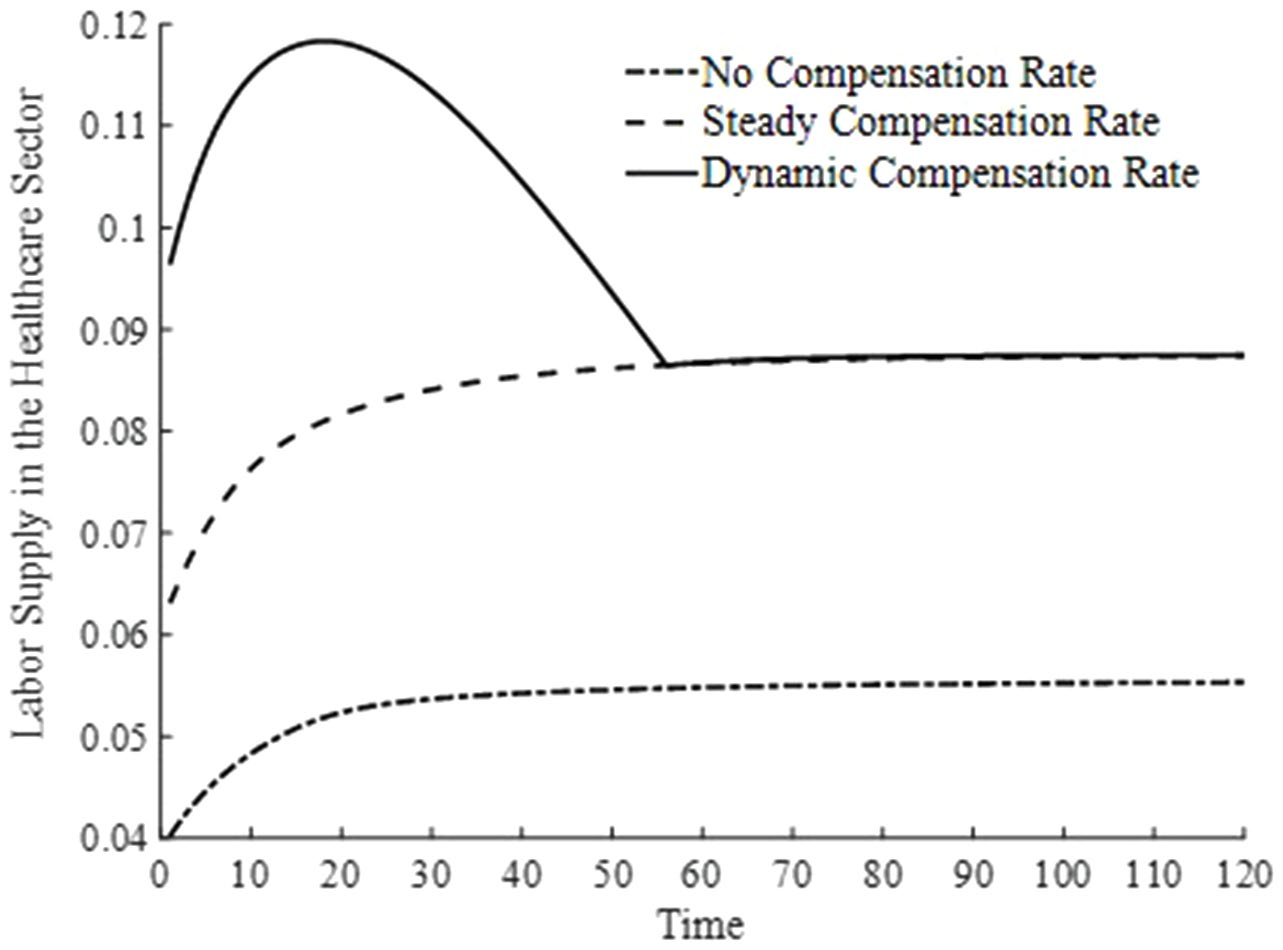
Labor supply in the healthcare sector.

**Figure 11:**
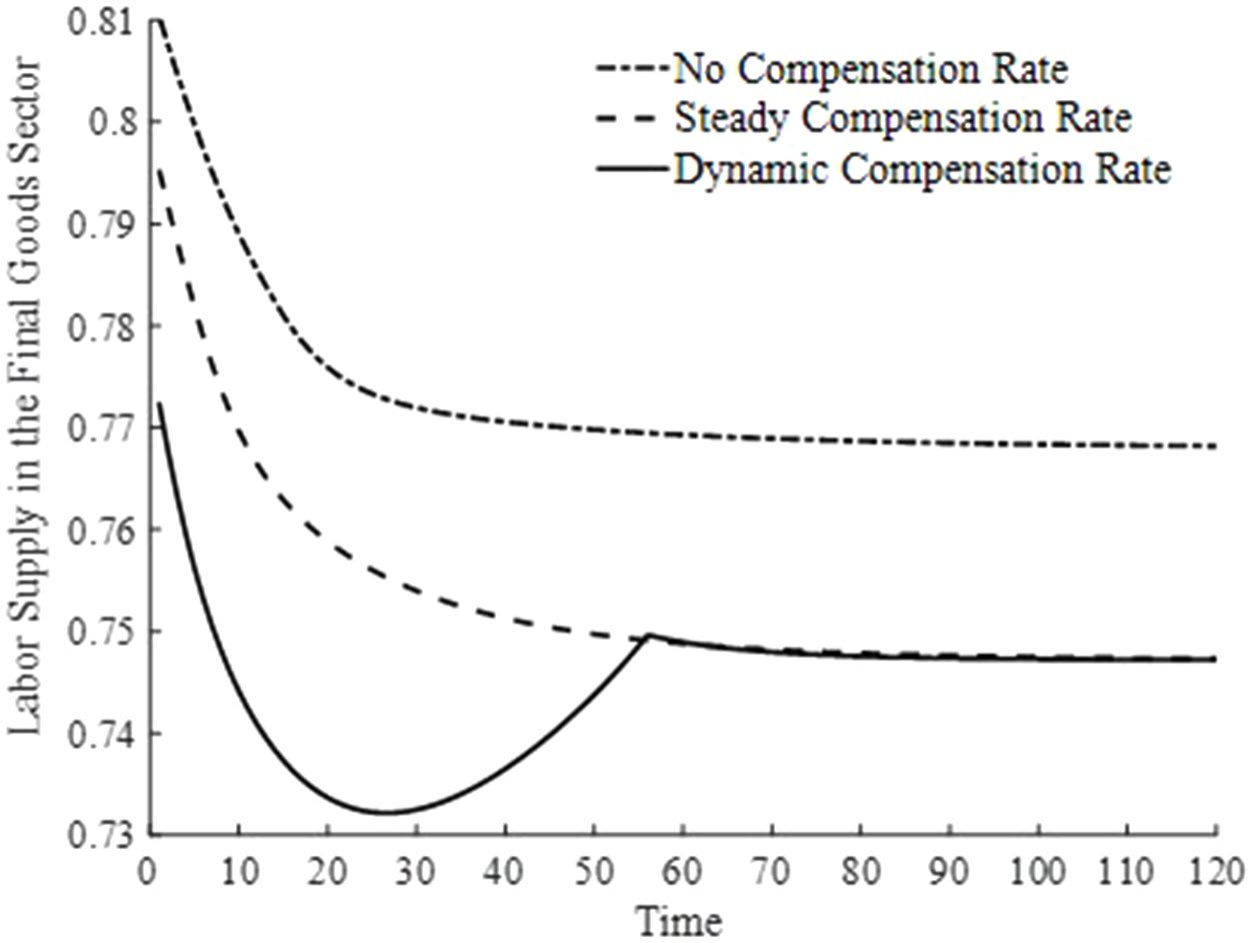
Labor supply in the final goods sector.

Figures 10, 11 illustrate the changes in labor supply among different sectors under three basic medical insurance compensation rates. Compared to the scenario without a basic medical insurance compensation rate, both the steady-state and dynamic compensation rates encourage labor to flow toward the healthcare sector. This shift is attributed to the rapid increase in medical consumption when a basic medical insurance compensation rate is in place. Businesses, aiming to maximize profits, have a higher demand for physical capital and labor, thereby encouraging labor to move toward the production of medical goods. Particularly under a dynamic basic medical insurance compensation rate, the labor supply shifts more swiftly toward the healthcare sector, leading to an increased production of medical products.

## 4 Discussion

This paper employs a neoclassical general equilibrium model to examine the impact of basic medical insurance compensation on health capital accumulation and economic growth. Under the objective of utility maximization, parameters related to the utility function play a crucial role in shaping the overall structure of the model. In particular, the estimation of certain parameters involves subjective factors tied to individual perceptions. Therefore, this section focuses on the sensitivity analysis of three parameters associated with the utility function—namely, the elasticity of substitution for leisure, the elasticity of substitution for medical goods demand, and the annual utility discount rate. Moreover, since the paper primarily concerns the dynamic paths of health capital accumulation and economic growth, we limit our discussion to how health capital and final goods output evolve over time under different parameter settings.

**Figure 12:**
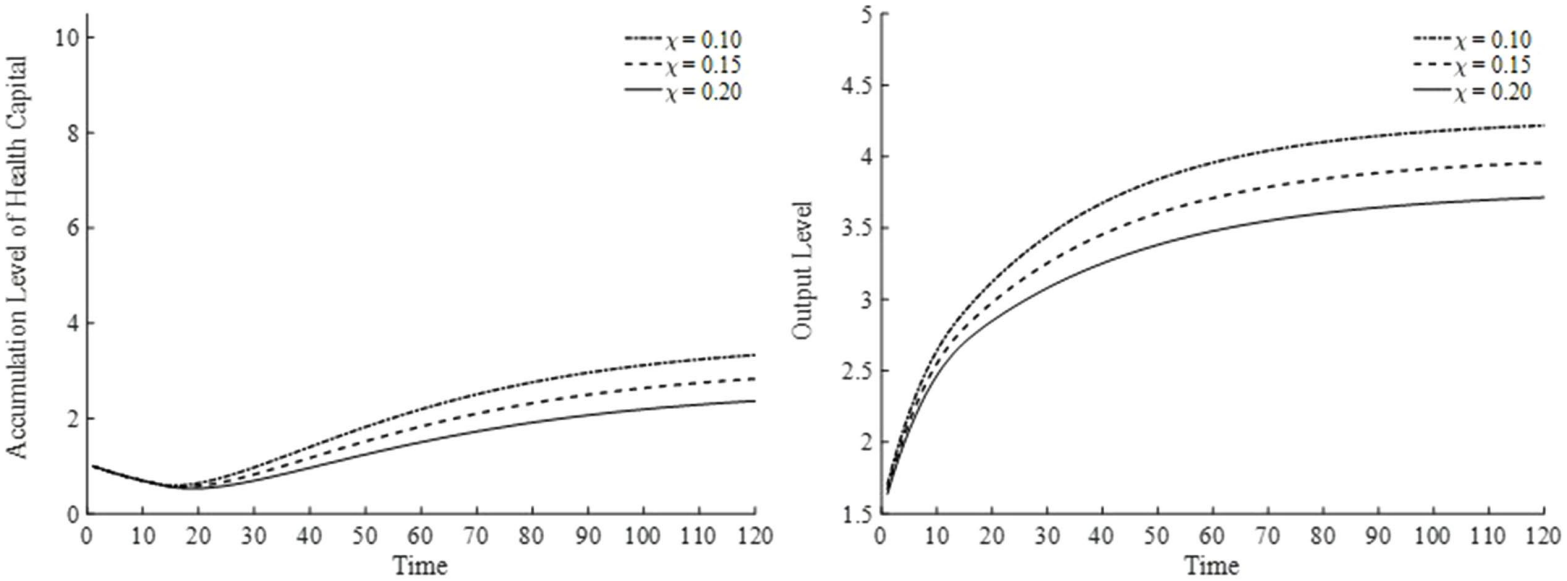
Health capital and output levels under different elasticities of substitution for leisure.

Regarding the elasticity of substitution for leisure, we consider three values: 0.10, 0.15, and 0.20. The results are presented in Figure 12 (the left panel depicts changes in health capital, and the right panel depicts changes in output). As shown in Figure 12, when the elasticity of substitution for leisure increases, leisure occupies a higher proportion in the household’s overall utility. Consequently, households spend more time on leisure and significantly reduce their labor input. This reduction in labor leads to pronounced decreases in both the health capital stock and output in the final goods sector.

**Figure 13:**
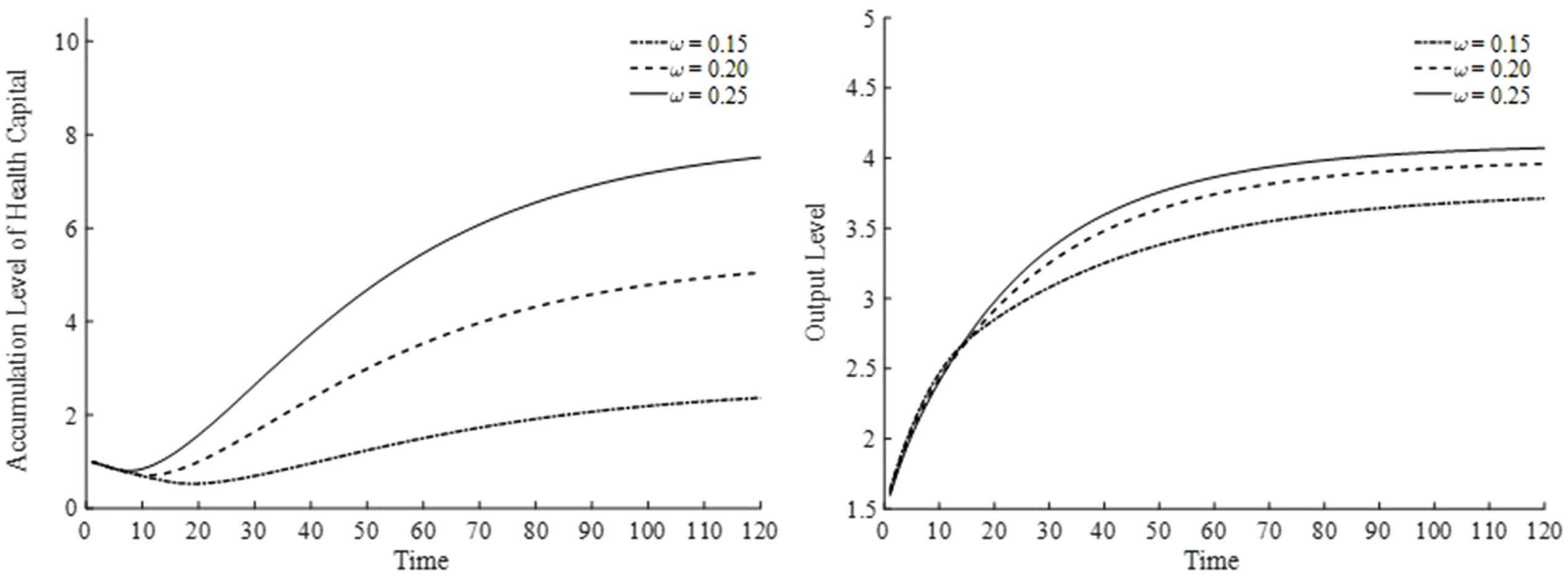
Health capital and output levels under different elasticities of substitution for medical goods.

With respect to the elasticity of substitution for medical goods demand, we select three values: 0.15, 0.20, and 0.25. The results are displayed in Figure 13. As the elasticity of substitution for medical goods demand rises, the utility share of medical goods consumption increases, indicating that households place greater value on medical services and therefore consume more medical products to improve their health. From the perspective of the overall economy, industrial structure gradually shifts, directing more capital and labor toward the medical goods sector. Consequently, as the health capital stock grows, the total factor productivity of the final goods sector also improves, contributing to a more robust economy. As illustrated in Figure 13, households become more inclined to invest in health, accumulate health capital, and boost output levels.

**Figure 14:**
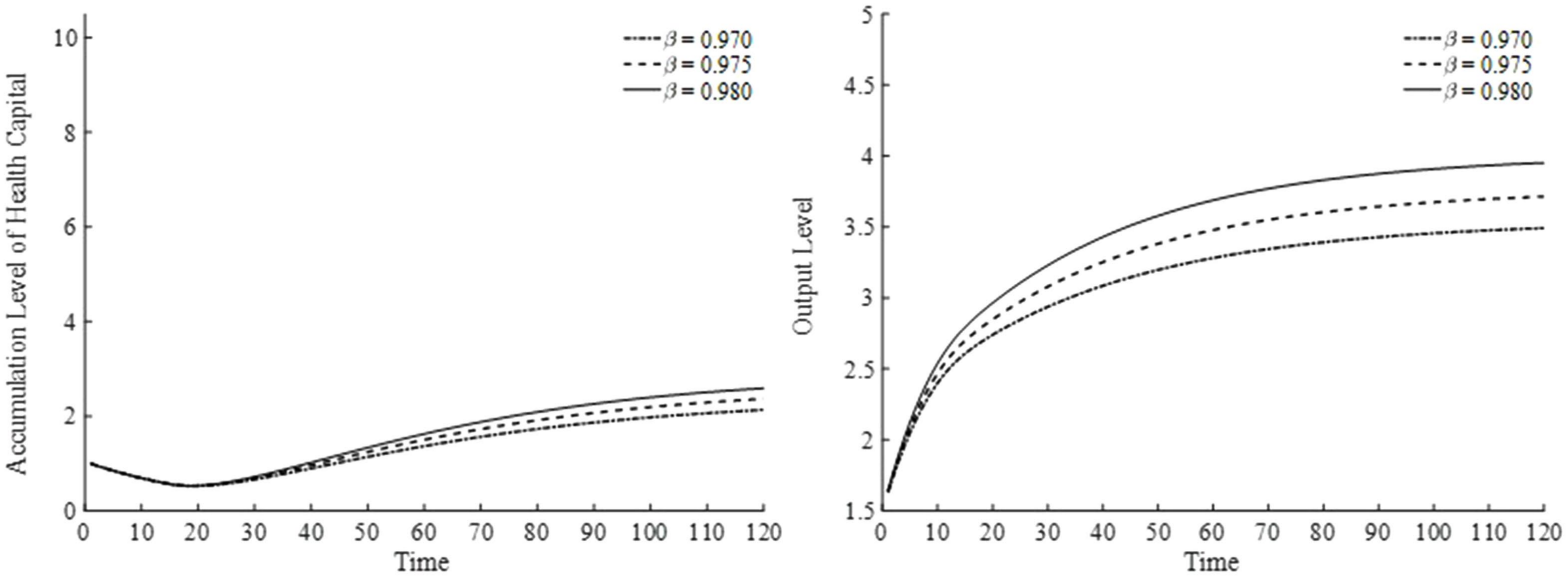
Health capital and output levels under different utility discount rates.

Lastly, for the annual utility discount rate, we choose three values—0.970, 0.975, and 0.980—and present the results in Figure 14. A higher annual utility discount rate raises the present value of future utility, indicating that households place greater emphasis on future consumption. Thus, as shown in Figure 14, households continuously accumulate health capital, thereby enhancing output over time.

In our model, we assume that medical products are consumed exclusively by households. First, because medical products are used solely for household medical consumption, the output of the medical sector is closely tied to the accumulation of health capital. This implies that as long as households value their personal health, not only does the medical sector expand, but the final goods sector’s output also rises. Second, the exclusive focus on household medical consumption means that medical products unrelated to non-medical household consumption are excluded, limiting a fully accurate depiction of the broader economic system. In particular, within a general equilibrium framework, the more sectors there are, the more challenging it becomes to thoroughly capture inter-sectoral linkages. Nevertheless, this simplification allows the analysis to concentrate on how medical consumption influences health capital accumulation. Third, under this assumption, while the model remains interpretable for settings involving multiple industries or sectors—or even scenarios with macro shocks—its applicability in those contexts is somewhat constrained. A more complex and comprehensive model would be needed to offer a fuller representation of the economic system in such scenarios.

In addition, by adopting the decreasing portion of an inverted-U shaped quadratic function to govern changes in the basic medical insurance compensation rate, policymakers aim to set a relatively high rate in the early stages of economic development, thereby accelerating health capital accumulation, alleviating households’ medical burden, and stimulating healthcare consumption. As the economy converges toward its steady state, the compensation rate is gradually reduced. On one hand, this helps curtail resource waste due to excessive medical treatment and prevents the crowding out of non-medical consumption. On the other hand, it incentivizes labor to transition from the healthcare sector to other productive sectors, thereby optimizing resource allocation.

From a theoretical standpoint, this design dynamically aligns a country’s stage of economic development with its health investment needs, thus balancing short-term improvements in health outcomes with long-term economic growth. In practice, however, policymakers must take precautions against potential welfare disparities stemming from uneven regional development—for instance, underdeveloped areas might risk falling into a health-poverty trap if compensation rates decline too rapidly. Moreover, precise fiscal actuarial assessments, enhanced tiered healthcare delivery systems, and robust public outreach initiatives are essential. These measures mitigate barriers posed by vested interests (e.g., reliance on hospital income) and reduce public misinterpretation of “welfare cuts,” thereby ensuring a smoother implementation of the policy.

This paper inevitably has certain limitations. Compared with the real world, the model presented here is simplified, and thus it cannot fully depict all facets of a real-world economy. However, our primary objective is to analyze the impact of basic medical insurance compensation on health capital accumulation and economic growth; hence, our focus is on this particular phenomenon. At the same time, our study is largely theoretical and does not include empirical testing. In future work, we will employ real-world data to further validate our theoretical findings. Additionally, while our analysis is grounded in the context of China’s economy, it provides insights that could be adapted—with caution—to other countries. Directly applying our methods or conclusions elsewhere would require adjustments reflecting the unique characteristics of each country.

## 5 Conclusion

This paper integrates health capital and economic growth into the neoclassical model endogenously, considering the positive externalities of health capital on production efficiency. Utilizing the Shooting algorithm, it calculates the equilibrium numerical solutions for various macroeconomic variables along the saddle path from initial levels of physical and health capital to the steady state. It analyzes the interaction between health capital and economic growth under a dynamic basic medical insurance compensation rate. The main conclusion is that a progressively decreasing basic medical insurance compensation rate facilitates the rapid accumulation of health capital, thereby propelling economic growth. Through the leverage of insurance, the dynamic basic medical insurance compensation rate not only improves health levels and promotes economic growth but also maximizes the external economic effects of health capital accumulation and the welfare level along the entire saddle path. Furthermore, under the influence of basic medical insurance compensation, there is an increase in labor supply throughout the saddle path, with a more significant portion of labor supply time flowing toward the healthcare sector.

In contrast, under the absence of a basic medical insurance compensation policy, since health capital is not incorporated into the optimization decisions, economic growth struggles to effectively promote medical consumption, and the positive externalities of health capital cannot be maximized to enhance production efficiency and improve the level of social welfare. A steady-state basic medical insurance compensation rate still fails to effectively and swiftly facilitate the accumulation of health capital along the saddle path, resulting in the welfare on the economic growth path remaining at a lower level continuously.

As the role of health capital in promoting economic growth strengthens, spontaneous medical consumption lacks effectiveness in accumulating health capital. Reliance solely on a steady-state, unchanging basic medical insurance compensation mechanism falls short of meeting the people’s needs for improved health levels and economic growth. It is imperative for the government to set a basic medical insurance compensation rate higher than the steady state in the early stages of economic development to match the needs of economic progress. As the economy continues to develop, appropriately reducing the basic medical insurance compensation rate can prevent the issue of excessive medical consumption leading to a decrease in non-medical consumption levels, achieving scientific development and continuous improvement in social welfare.

In fact, the effective implementation of a dynamic basic medical insurance (BMI) compensation policy requires overcoming multiple practical challenges and adapting to the unique characteristics of China’s healthcare system.

First, from an administrative perspective, adjusting compensation rates dynamically involves cooperation among multiple government departments—health insurance, finance, and public health—potentially leading to policy fragmentation and discrepancies in local implementation. To address this issue, we recommend establishing a cross-departmental coordination mechanism at the central level, integrating the revision of insurance coverage, fiscal budget allocations, and healthcare service pricing powers to ensure consistency in policy objectives. At the same time, a differentiated regional compensation framework is needed: based on a nationally determined baseline compensation rate, provincial authorities could adjust the rate within a fixed interval (e.g., ±20%) according to local economic development and healthcare resource availability, using central fiscal transfers to bridge interregional welfare disparities. For instance, in underdeveloped central and western regions, one might extend the phase-down period for compensation rates and simultaneously strengthen investment in primary healthcare facilities to avoid exacerbating health inequities.

Second, mitigating fiscal sustainability risks requires establishing a dynamic actuarial model and diversified financing mechanisms. Drawing on demographic changes, shifts in disease profiles, and economic growth forecasts, policymakers could set a threshold for health insurance fund balances (e.g., triggering a compensation rate reduction if the surplus falls below 15%), and explore incorporating state-owned enterprise profits or earmarked tobacco taxes into the health insurance fund. Meanwhile, it is crucial to address resistance from vested interests. For example, to manage the potential decrease in revenue for public hospitals, implementing DRG (Diagnosis-Related Group) or DIP (Diagnosis-Intervention Packet) payment reforms can shift the focus from fee-for-service toward payment based on health outcomes. Similarly, to counter insufficient innovation incentives among pharmaceutical companies, tax breaks can encourage the development of higher value-added medications, reducing reliance on BMI compensation rates.

Third, on a technical level, policy implementation should be closely integrated with the existing healthcare system. On the one hand, linking dynamic compensation rates with the tiered healthcare delivery system is essential: higher compensation rates (e.g., 90%) may be offered to primary healthcare facilities, while gradually reducing rates for tertiary hospitals to a stable level (e.g., 70%), thereby promoting the decentralization of medical resources and optimizing the labor supply structure. On the other hand, leveraging digital technologies can enhance policy precision. For instance, building an intelligent health insurance monitoring platform that uses big data to identify excessive medical practices and dynamically adjust individual compensation rates can improve cost-effectiveness. Additionally, introducing personal health points accounts—tying preventive care behaviors (e.g., regular checkups, health management) to compensation entitlements—would foster public acceptance of gradually reduced compensation rates.

Finally, given China’s urban–rural dual structure and aging population, the policy design must incorporate adaptive innovations. Concerning urban–rural integration, a 3–5-year extension of the compensation rate adjustment period could be granted to participants in the New Rural Cooperative Medical System, relative to the Urban Resident Basic Medical Insurance scheme, while accelerating the unification of insurance coverage and reimbursement ratios across regions to narrow institutional welfare gaps. In addressing the challenges of an aging society, a “high-age exemption” mechanism could suspend compensation rate reductions for individuals over 70 years old, with long-term care insurance helping cover medical care costs. Furthermore, a “time bank” mutual-aid model could incentivize relatively younger, healthier seniors to participate in community health services in exchange for future BMI compensation rights, thereby facilitating intergenerational resource redistribution.

## Data Availability

The original contributions presented in the study are included in the article/supplementary material, further inquiries can be directed to the corresponding author.

## Footnotes

^1^The sector encompasses both Class A drugs (e.g., basic medications for chronic diseases, anti-infective drugs, and essential therapeutic drugs) and Class B drugs (e.g., targeted cancer therapies, advanced medications for chronic disease management, and immunotherapies). In China, medical products eligible for reimbursement are primarily therapeutic, and this paper focuses on the externalities of medical insurance compensation. Therefore, the model is primarily concerned with therapeutic medical products.

^2^Finkelstein et al. (25) empirically validated that health status affects marginal utility and incorporated the demand for health goods into the utility function.

^3^As noted above, we maintain that the basic medical insurance compensation rate should decrease non-linearly and be highest in the initial period. Accordingly, based on the characteristics of a single-variable quadratic function, we set b=0. Empirically, China’s basic medical insurance compensation rate currently averages between 60 and 70%. Thus, we specify the initial compensation rate to be approximately 65%. Moreover, when basic medical insurance compensation is in place, the economy’s steady state is reached around year 55. From a utility-maximizing perspective, calculations indicate that the optimal steady-state compensation rate is roughly 41%. Therefore, we obtain $a=-0.8033\times 10^{5}$ and c=0.648.

## References

[1] Grossman M. On the concept of health capital and the demand for health. J Polit Econ. (1972) 80:223–55. doi: 10.1086/259880

[2] Ehrlic I Lui FT. Intergenerational trade, longevity, and economic growth. J Polit Econ. (1991) 99:1029–59. doi: 10.1086/261788

[3] Bloom DE Canning D Sevilla J. The effect of health on economic growth: a production function approach. World Dev. (2004) 32:1–13. doi: 10.1016/j.worlddev.2003.07.002

[4] Barro RJ. Inflation and economic growth. Ann Econ Financ. (2013) 14:305–42.

[5] Kelly M. Health capital accumulation, health insurance, and aggregate outcomes: a neoclassical approach. J Macroecon. (2017) 52:1–22. doi: 10.1016/j.jmacro.2017.02.003

[6] Atolia M Papageorgiou C Turnovsky SJ. Taxation and public health investment: policy choices and tradeoffs. Macroecon Dyn. (2021) 25:426–61. doi: 10.1017/S1365100519000245, PMID: 40166671

[7] Halliday TJ He H Ning L Zhang H. Health investment over the life-cycle. Macroecon Dyn. (2019) 23:178–215. doi: 10.1017/S1365100516001152

[8] Agénor PR. Health and infrastructure in a model of endogenous growth. J Macroecon. (2008) 30:1407–22. doi: 10.1016/j.jmacro.2008.04.003, PMID: 40179927

[9] Liu K Prommawin B Schroyen F. Health insurance, agricultural production and investments. J Health Econ. (2024) 97:102918. doi: 10.1016/j.jhealeco.2024.102918, PMID: 39180871

[10] Liu GG Dow WH Fu AZ Lance P. Income productivity in China: on the role of health. J Health Econ. (2008) 27:27–44. doi: 10.1016/j.jhealeco.2007.05.001, PMID: 17645977

[11] Bai CE Wu B. Health insurance and consumption: evidence from China's new cooperative medical scheme. J Comp Econ. (2014) 42:450–69. doi: 10.1016/j.jce.2013.07.005

[12] Liu K. Insuring against health shocks: health insurance and household choices. J Health Econ. (2016) 46:16–32. doi: 10.1016/j.jhealeco.2016.01.002, PMID: 26836108

[13] Zhou M Zhao S Zhao Z. Gender differences in health insurance coverage in China. Int J Equity Health. (2021) 20:52–64. doi: 10.1186/s12939-021-01383-9, PMID: 33526037 PMC7852118

[14] Lu JR Leung GM Kwon S Tin KYK Van Doorslaer E O’Donnell O. Horizontal equity in health care utilization evidence from three high-income Asian economies. Soc Sci Med. (2007) 64:199–212. doi: 10.1016/j.socscimed.2006.08.033, PMID: 17014944

[15] Boutin D Petifour L Allard Y Kountoubré S Ridde V. Comprehensive assessment of the impact of mandatory community-based health Insurance in Burkina Faso. Soc Sci Med. (2025) 371:117870. doi: 10.1016/j.socscimed.2025.117870, PMID: 40064144

[16] Cook AC Sirmans ET Stype A. Medical cannabis laws lower individual market health insurance premiums. Int J Drug Policy. (2023) 119:104143. doi: 10.1016/j.drugpo.2023.104143, PMID: 37572391

[17] Frankovic I Kuhn M. Health insurance, endogenous medical progress, health expenditure growth, and welfare. J Health Econ. (2023) 87:102717. doi: 10.1016/j.jhealeco.2022.102717, PMID: 36638641

[18] Jung J Tran C. Social health insurance: a quantitative exploration. J Econ Dyn Control. (2022) 139:104374. doi: 10.1016/j.jedc.2022.104374, PMID: 40179927

[19] van Doorslaer E Masseria C Koolman X. Inequalities in access to medical care by income in developed countries. Can Med Assoc J. (2006) 174:177–83. doi: 10.1503/cmaj.050584, PMID: 16415462 PMC1329455

[20] van Doorslaer E Wagstaff A van der Burg H Christiansen T De Graeve D. Equity in the delivery of health care in Europe and the US. J Health Econ. (2000) 19:553–83. doi: 10.1016/S0167-6296(00)00050-3, PMID: 11184794

[21] Zhou XH Yang XJ. Medical insurance, vulnerability to poverty, and wealth inequality. Front Public Health. (2024) 12:1286549. doi: 10.3389/fpubh.2024.1286549, PMID: 38476487 PMC10927954

[22] Finkelstein A Hendren N Shepard M. Subsidizing health insurance for low-income adults: evidence from Massachusetts. Am Econ Rev. (2019) 109:1530–67. doi: 10.1257/aer.20171455, PMID: 30990593

[23] Hamid SA Roberts J Mosley P. Can micro health insurance reduce poverty? Evidence from Bangladesh. J Risk Insur. (2011) 78:57–82. doi: 10.1111/j.1539-6975.2010.01402.x

[24] Herrendorf B Rogerson R Valentinyi A. Two perspectives on preferences and structural transformation. Am Econ Rev. (2013) 103:2752–89. doi: 10.1257/aer.103.7.2752

[25] Finkelstein A Luttmer E Notowidigdo MJ. What good is wealth without health? The effect of health on the marginal utility of consumption. J Eur Econ Assoc. (2013) 11:221–58. doi: 10.1111/j.1542-4774.2012.01101.x

[26] Huang K Huffman GW. Unemployment and welfare implications of the current U.S. tax treatment of employer-provided medical insurance. Macroecon Dyn. (2014) 18:1547–80. doi: 10.1017/S1365100513000023

[27] Fan QQ Zhang TB. A study of environmental regulations and pollution abatement mechanism on china's economic growth path. J World Econ. (2018) 41:171–92. doi: 10.19985/j.cnki.cassjwe.2018.08.009

[28] Zhou XH Fan QQ. Mechanism of carbon intensity reduction and optimization Design of its Industrial Allocation. J World Econ. (2016) 39:168–92. doi: 10.19985/j.cnki.cassjwe.2016.07.009

[29] Leukhina OM Turnovsky SJ. Population size effects in the structural development of England. Am Econ J Macroecon. (2016) 8:195–229. doi: 10.1257/mac.20140032, PMID: 40153406

[30] Fan QQ Zhou XH Zhang TB. Externalities of dynamic environmental taxation, paths of accumulative pollution and long-term economic growth. Econ Res J. (2016) 51:116–28.

